# Osteogenic Activities of Trifolirhizin as a Bioactive Compound for the Differentiation of Osteogenic Cells

**DOI:** 10.3390/ijms242317103

**Published:** 2023-12-04

**Authors:** Hyung-Mun Yun, Mi Hyeon Cho, Hoibin Jeong, Soo Hyun Kim, Yun Hee Jeong, Kyung-Ran Park

**Affiliations:** 1 Department of Oral and Maxillofacial Pathology, School of Dentistry, Kyung Hee University, Seoul 02447, Republic of Korea; 2 Korea Basic Science Institute (KBSI), Seoul 02841, Republic of Korea; mhcho1@kbsi.re.kr (M.H.C.); hoibinjeong4@kbsi.re.kr (H.J.); 3 National Development Institute for Korean Medicine, Gyeongsan 38540, Republic of Korea; beluga81@nikom.or.kr (S.H.K.); jyh@nikom.or.kr (Y.H.J.); 4 Korea Basic Science Institute (KBSI), Gwangju 61751, Republic of Korea

**Keywords:** bone, differentiation, osteoblast, RUNX2, *Sophora flavescens*, trifolirhizin

## Abstract

Plant extracts are widely used as traditional medicines. *Sophora flavescens* Aiton-derived natural compounds exert various beneficial effects, such as anti-inflammatory, anticancer, antioxidant, and antiregenerative activities, through their bioactive compounds, including flavonoids and alkaloids. In the present study, we investigated the biological effects of an *S. flavescens*-derived flavonoid, trifolirhizin (trifol), on the stimulation of osteogenic processes during osteoblast differentiation. Trifol (>98% purity) was successfully isolated from the root of *S. flavescens* and characterized. Trifol did not exhibit cellular toxicity in osteogenic cells, but promoted alkaline phosphatase (ALP) staining and activity, with enhanced expression of the osteoblast differentiation markers, including *Alp*, *ColI*, and *Bsp*. Trifol induced nuclear runt-related transcription factor 2 (RUNX2) expression during the differentiation of osteogenic cells, and concomitantly stimulated the major osteogenic signaling proteins, including GSK3β, β-catenin, and Smad1/5/8. Among the mitogen-activated protein kinases (MAPKs), Trifol activated JNK, but not ERK1/2 and p38. Trifol also increased the osteoblast-mediated bone-forming phenotypes, including transmigration, F-actin polymerization, and mineral apposition, during osteoblast differentiation. Overall, trifol exhibits bioactive activities related to osteogenic processes via differentiation, migration, and mineralization. Collectively, these results suggest that trifol may serve as an effective phytomedicine for bone diseases such as osteoporosis.

## 1. Introduction

Dysregulation in bone metabolism and remodeling are features of serious bone diseases, such as osteoporosis and periodontitis [1]. Dysfunction in the proliferation, migration, and differentiation of osteogenic cells leads to deficient and excessive bone formation [2]. Mesenchymal progenitor cells differentiate into osteoblasts [3]. Mesenchymal progenitor cells differentiate into osteoblasts [3]. Osteoblasts are bone-forming cells required for bone synthesis and mineralization during bone development and remodeling [3,4]. Mesenchymal progenitor cells undergo differentiation into osteocytes, and the bone matrix eventually surrounds the osteocytes and becomes calcified [5,6,7]. Osteoblast differentiation is induced and regulated by multicellular signaling. The Wnt family member 3a (Wnt3a), bone morphogenetic protein 2 (BMP2), and mitogen-activated protein kinase (MAPKs)-mediated signaling pathways are mainly responsible for controlling the nuclear expression and activity of runt-related transcription factor 2 (RUNX2), which is a master protein for osteoblast differentiation [8,9,10]. Consequently, osteoblasts physiologically produce and secrete different molecules, such as collagen, growth factors, alkaline phosphatase (ALP), and numerous non-collagenous proteins. Osteoblast dysfunction is associated with pathological bone loss [5,6].

Plant extracts containing bioactive compounds, such as flavonoids, alkaloids, and terpenoids, are used to treat and prevent various diseases in traditional medicines; these compounds exert therapeutic effects by stimulating multicellular signaling and biological mechanisms [11,12]. Plant-derived drugs have fewer side effects than chemically synthesized drugs, and are suitable for long-term use [11,13]. Therefore, plant-derived medicines are valuable for the research and development of modern medications. Many medications have been developed using plant extracts [14]. *Sophora flavescens* Aiton (also called Ku Shen in Republic of Korea) has a wide range of biological activities. Therefore, *S. flavescens* is valued in both traditional and modern medicines as a significant resource [15]. *S. flavescens* exerts its biological activities through its chemical constituents, particularly flavonoids (mainly trifolirhizin (trifol), M = maackiain, kushenol I, kurarinone, and sophoraflavanone G) and alkaloids (mainly matrine, oxymatrine, sophorine, and oxysophoridine) [16,17,18,19]. Flavonoids from *S. flavescens* have various beneficial effects, such as anti-inflammatory, anti-cancer, anti-bacterial, anti-diabetic, and anti-arthritic activities [20,21,22]. Maackiain decreases osteoclastogenesis through the RANKL-mediated signaling pathway, and 8-Prenylkaempferol increases osteoblast maturation through the BMP2-Smad1/5/8- and BMP2-p38-mediated pathways [23,24]. Trifol, a flavonoid isolated from *S. flavescens*, is a bioactive compound used in traditional Chinese medicine (Xian-Lian-Ke-Li) for cancer prevention and has anti-inflammatory, anti-proliferative, anti-cancer, and skin-whitening effects [25,26,27,28,29]. However, the effects of trifol on osteoblast differentiation have not been reported. We hypothesize that trifol has beneficial effects on osteoblast differentiation and maturation.

In the present study, trifol (>98% purity) was isolated from the roots of *S. flavescens*. In this study, the pharmacological effects of trifol on osteogenic processes, intracellular signaling, and matrix mineralization during the differentiation of osteogenic MC3T3-E1 cells in vitro were evaluated.

## 2. Results

### 2.1. Isolation and Characterization of Trifol from the Root of Sophora flavescens

**Figure 1:**
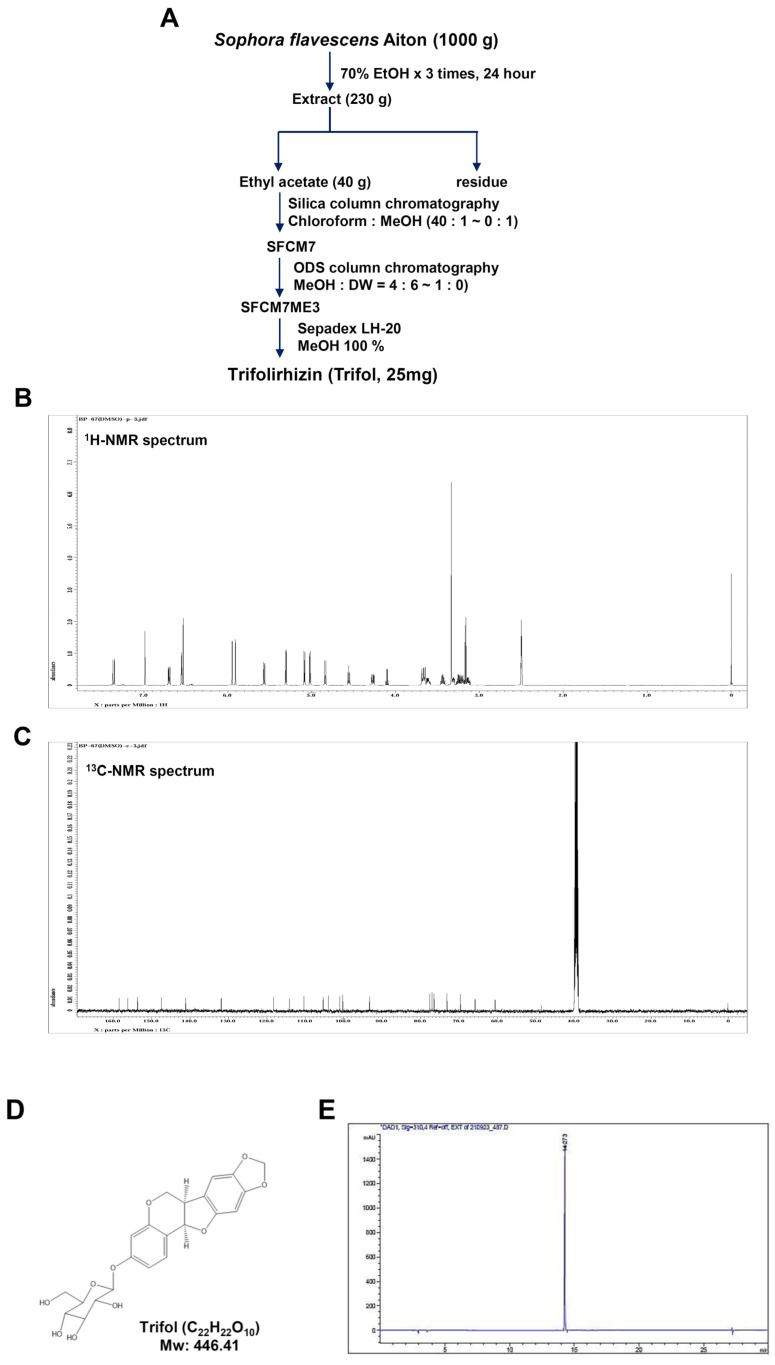
Purification and characterization of trifol from the dried root of *S. flavescens*. (**A**) Flowchart for the isolation of trifol. (**B**,**C**) ^1^H-NMR (500 MHz, DMSO-d_6_) spectrum (**B**) and ^13^C-NMR (125 MHz, DMSO-d_6_) spectrum (**C**). (**D**,**E**) Chemical structure (**D**) and HPLC (**E**) of trifol (C_22_H_22_O_10_, purity: >98% purity).

Trifol was purified from the dried roots of *Sophora flavescens* (Figure 1A). Trifol was characterized using nuclear magnetic resonance (NMR). ^1^H-NMR (500 MHz, DMSO-d_6_) δ 7.30 (1H, d, J = 8.5 Hz, H-l), 6.99 (1H, s, H-7), 6.70 (1H, dd, J = 8.5 and 1.8 Hz, H-2), 6.50 (1H, d, J = 1.8 Hz, H-4), 6.49 (1H, s, H-10), 5.90 (2H, d, J = 12.8 Hz H-CH2O), 5.52 (1H, d, J = 6.8 Hz, H-11a), 5.02 (1H, d, J = 7.2 Hz, H-l’), 4.27 (1H, dd, J = 4.0 and 10.5 Hz, H-6), 3.62 (1H, dd, J = 4.2 and 7.12 Hz, H-6), 3.32–3.43 (2H, m, H-5’, 6’), 3.15–3.28 (3H, m, H-2’, 3’, 4’) (Figure 1B). ^13^C-NMR (125 MHz, DMSO-d_6_) δ 159.3 (C-3), 157.0 (C-4a), 154.5 (C-10a), 148.3 (C-9), 142.0 (C-8), 132.8 (C-1), 119.1 (C-6b), 115.0 (C-11b), 111.8 (C-2), 106.2 (C-7), 104.8 (C-4), 101.9 (C-1), 101.1 (-OCH2O-), 94.1 (C-10), 78.5 (C-11a), 77.9 (C-3), 77.4 (C-5), 74.0 (C-2), 70.5 (C-4), 66.7 (C-6), 61.5 (C-6), 41.3 (C-6a) (Figure 1C). The chemical structure and high-performance liquid chromatography (HPLC) results (colorless powder, molecular formula: C_22_H_22_O_10_, purity > 98%) are shown in Figure 1D,E.

### 2.2. Trifol Increases Osteoblast Differentiation and the Levels of Osteogenic Marker Genes

**Figure 2:**
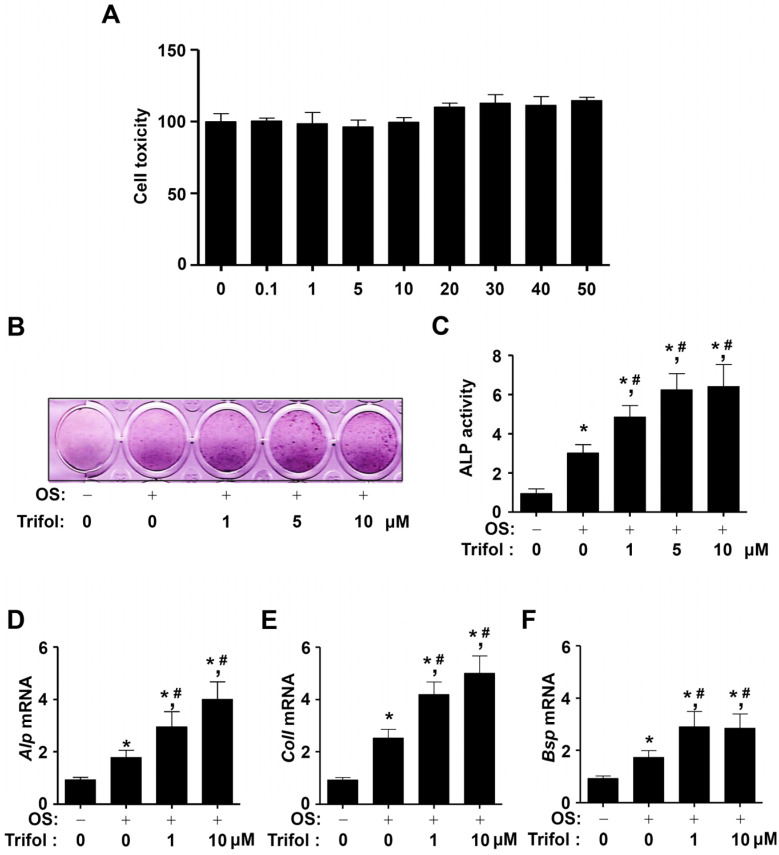
Cytotoxicity and osteoblast differentiation due to trifol in osteogenic cells. (**A**) Osteogenic cells were treated with the indicated concentrations of trifol for 24 h. Cell toxicity (%) was analyzed using an MTT assay. (**B**,**C**) Cells were treated in OS with the indicated concentrations of trifol for 7 days. ALP levels were measured using ALP staining (**B**) and activity (**C**) assays. (**D**–**F**) Cells were treated in OS with the indicated concentrations of trifol for 7 days. The mRNA levels of *Alp* (**D**), *ColI* (**E**), and *Bsp* (**F**) were detected using PCR; the relative fold is shown as a bar graph. OS: osteogenic supplement medium containing 50 μg/mL L-ascorbic acid and 10 mM β-glycerophosphate. Data are expressed as the mean ± SEM. * *p* < 0.05: statistically significant compared to the control. #, *p* < 0.05: statistically significant compared to OS. Data represent the results of three experiments.

We investigated whether trifol induced cytotoxicity and osteoblast differentiation in osteogenic MC3T3-E1 cells. In the MTT assay, trifol exerted no cytotoxicity at concentrations up to 50 μM (Figure 2A). As a result, low concentrations (1–10 μM) of trifol, which were found to be non-cytotoxic, were used in subsequent experiments. ALP activity is the main phenotypic marker of osteoblastic differentiation. The effect of trifol on the differentiation of osteogenic cells was determined based on ALP activity. In the ALP staining and activity assays, ALP activity was found to increase in trifol-treated cells compared to cells treated with OS alone (Figure 2B,C). The osteogenic effects of trifol were validated by detecting osteogenic marker genes in trifol-treated cells. Trifol increased the mRNA levels of *Alp*, collagen type I (*ColI*), and bone sialoprotein (*Bsp*) compared to OS alone (Figure 2D–F). These data suggest that trifol exhibits biological activity in osteogenic cells.

### 2.3. Trifol Enhances Osteogenic Phenotype and Mineralization in Osteoblast Differentiation

**Figure 3:**
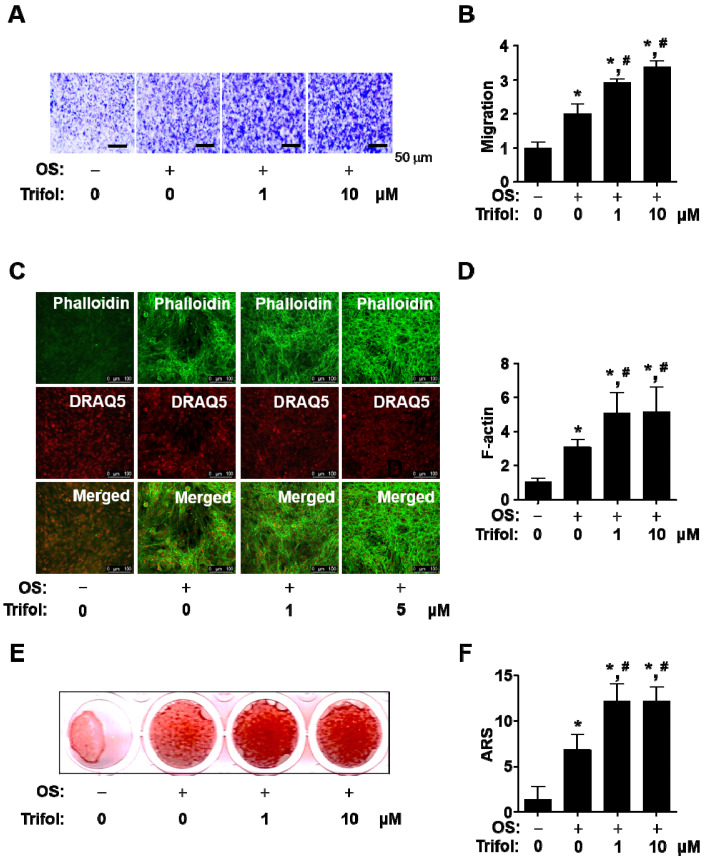
Promotion of osteogenic phenotypes and maturation by trifol in osteogenic cells. (**A**,**B**) Cells were treated in OS with the indicated concentrations of trifol for 3 days, and cell migration was measured using a Matrigel-coated membrane in a Boyden chamber. Scale bar: 50 μm (**A**); the relative levels are displayed as a bar graph (**B**). (**C**,**D**) At 3 days, F-actin polymerization was measured using phalloidin (green) and DRAQ5 (red) staining assay. Scale bar: 100 μm. (**C**); the relative levels are displayed as a bar graph (**D**). (**E**,**F**) At 14 days, mineralization was visualized using ARS staining (**E**); the relative levels are displayed as a bar graph (**F**). OS: osteogenic supplement medium containing 50 μg/mL L-ascorbic acid and 10 mM β-glycerophosphate. *, *p* < 0.05 compared with the control; #, *p* < 0.05 compared with OS. Data represent the results of three experiments.

Osteogenic processes induce migration and cytoskeletal changes, leading to osteoblast maturation and bone formation. Trifol was found to increase transmigration across the extracellular matrix (ECM) (Figure 3A,B). Thereafter, cytoskeletal changes were observed using an intravital multiphoton microscope (IMPM). Trifol significantly promoted morphological changes during osteoblast differentiation on Matrigel-coated culture plates (Figure 3C,D). Finally, we investigated whether trifol accelerated mineralization based on the accumulation of calcium deposits. Using alizarin red S (ARS) staining, trifol was found to increase mineralization during osteoblast maturation (Figure 3E,F). These data suggest that trifol induces bone-forming phenotypes, such as cell migration, morphological changes, and calcium deposits, during osteoblast differentiation.

### 2.4. Trifol Enhances Nuclear RUNX2 Expression and Osteogenic Signaling Proteins in Osteoblast Differentiation

**Figure 4:**
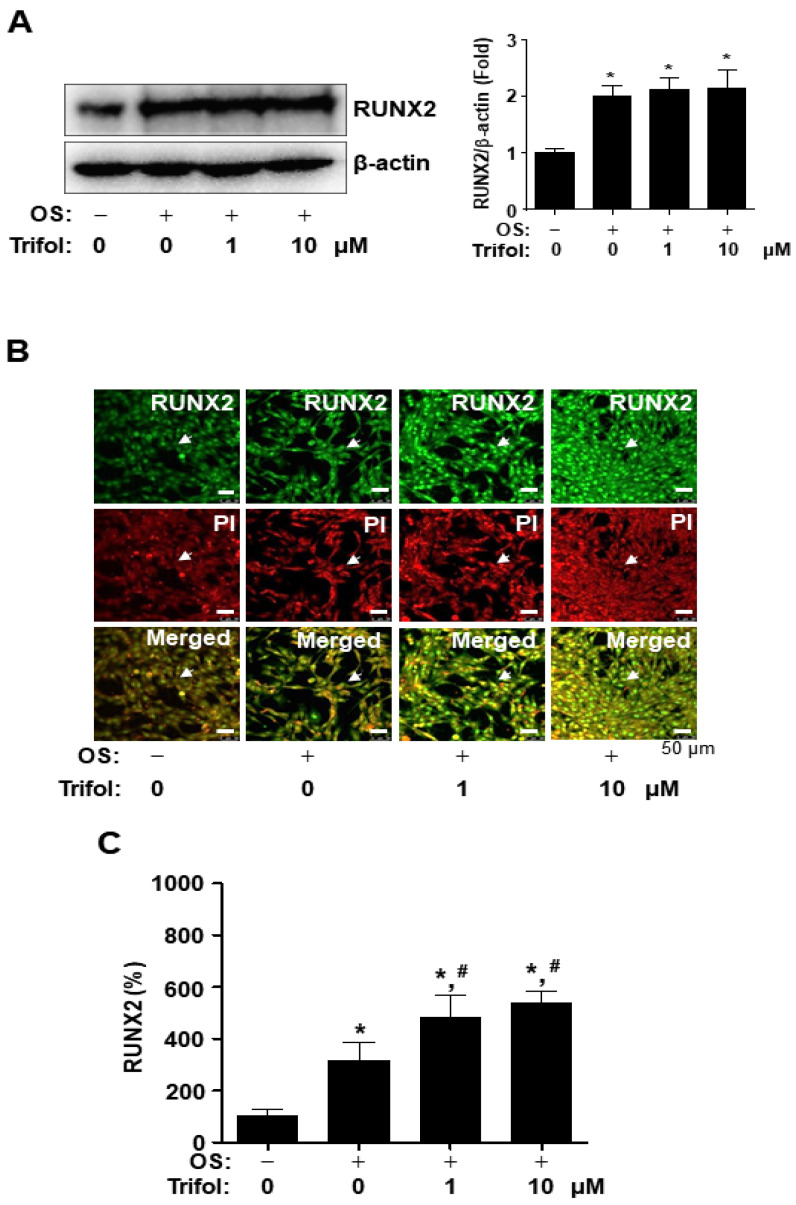
Expression and localization of RUNX2 due to trifol in osteogenic cells. (**A**) After osteogenic cells were cultured with OS for 3 days, protein levels of RUNX2 were detected using Western blot analysis, and the levels were standardized using the level of total β-actin, which served as a loading control. (**B**,**C**) At 3 days, RUNX2 levels in the nucleus were monitored via an immunofluorescence analysis with a RUNX2 antibody (green) and a nuclear marker, PI (red). The arrows indicate the nuclei. Scale bar: 50 μm (**B**). Relative levels are displayed in a bar graph (**C**). OS: osteogenic supplement medium containing 50 μg/mL L-ascorbic acid and 10 mM β-glycerophosphate. Data are expressed as the mean ± SEM. * *p* < 0.05: statistically significant compared to the control. #, *p* < 0.05: statistically significant compared to OS. Data represent the results of three experiments.

RUNX2 is a master transcription factor involved in osteogenic gene expression and differentiation. RUNX2 expression levels were determined using Western blot analysis. Trifol increased the total RUNX2 expression compared to the control, but did not alter the RUNX2 expression compared to OS (Figure 4A), suggesting that trifol increases the nuclear RUNX2 expression, but not total RUNX2 expression. The expression of RUNX2 was further examined by means of an immunofluorescence assay using PI, a nuclear marker. Nuclear RUNX2 levels were significantly enhanced in trifol-treated cells (Figure 4B).

**Figure 5:**
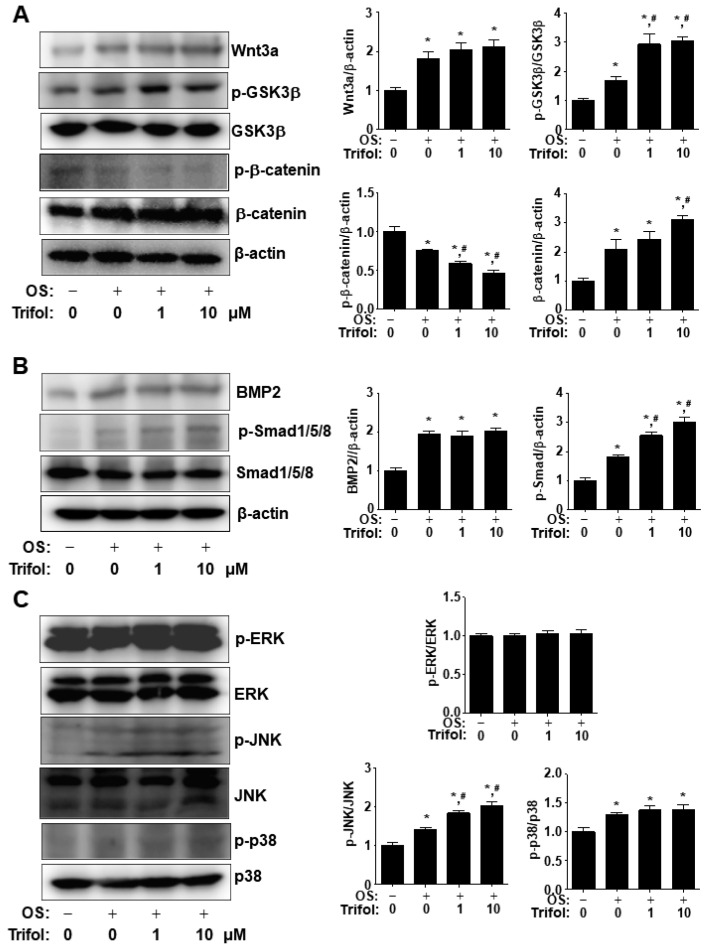
Stimulation of osteogenic signaling proteins by trifol in osteogenic cells. (**A**,**B**) After osteogenic cells were cultured with OS for 3 days, protein and phosphorylation levels of Wnt3a, p-GSK3β, GSK3β, p-β-catenin, and β-catenin (**A**), as well as BMP2, p-Smad1/5/8, and Smad1/5/8 (**B**), were detected using Western blot analysis. The levels were standardized using the level of total β-actin, which served as a loading control. (**C**) Phosphorylation levels of MAPKs, including ERK1/2, JNK, and p38, were detected using Western blot analysis. The levels were standardized using those of total ERK1/2, JNK, and p38, respectively, which served as loading controls. OS: osteogenic supplement medium containing 50 μg/mL L-ascorbic acid and 10 mM β-glycerophosphate. Data represent the results of three experiments. *, *p* < 0.05 compared with the control; #, *p* < 0.05 compared with OS. Data represent the results of three experiments.

We proceeded to investigate the cellular signaling proteins involved in nuclear RUNX2 expression. Compared with OS alone, trifol stimulated GSK3β phosphorylation, β-catenin dephosphorylation, β-catenin expression, and Smad1/5/8 phosphorylation, but did not affect Wnt3a or BMP2 expression (Figure 5A,B). In addition, the phosphorylation levels of JNK were significantly elevated in trifol-treated cells (Figure 5C); however, those of ERK1/2 and p38 remained unchanged in the trifol-treated cells (Figure 5C). These data suggest that trifol regulates the major osteogenic signaling proteins, p-GSK3β, β-catenin, p-Smad1/5/8, and p-JNK, to exert its biological activities in osteogenic cells.

### 2.5. Trifol Enhances Osteoblast Differentiation through the Stimulation of Smad1/5/8 and β-Catenin

**Figure 6:**
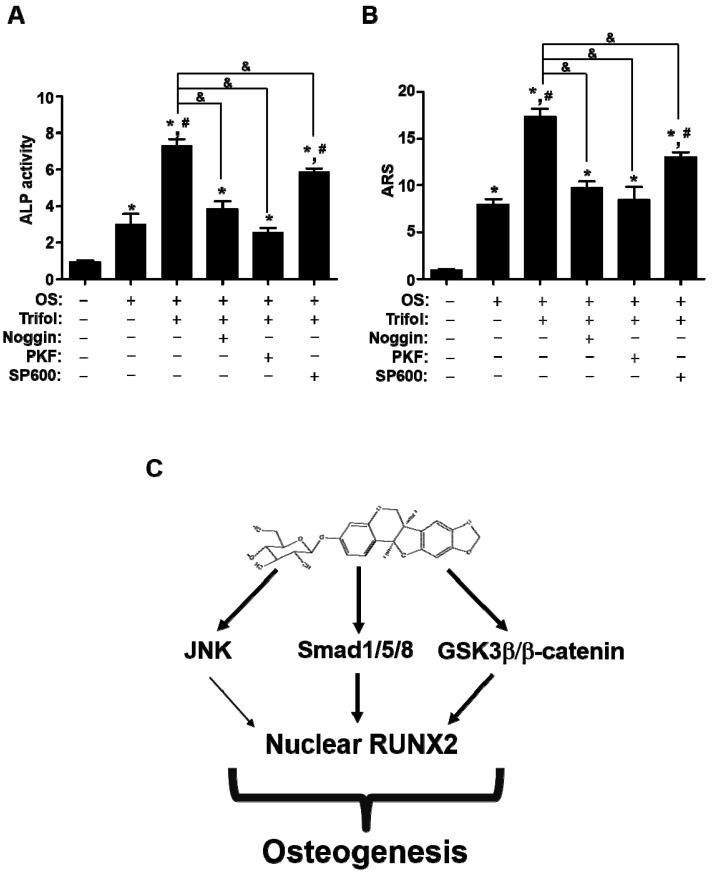
Trifol promotes osteoblast differentiation by stimulating osteogenic signaling proteins (**A**,**B**) After cells were treated in OS with trifol in the absence or presence of Noggin, PKF, and SP600. At 7 days, ALP levels were measured using ALP activity assay (**A**). At 14 days, mineralization was measured using ARS staining assay (**B**). (**C**) Schematic showing trifol-mediated osteoblast differentiation. OS: osteogenic supplement medium containing 50 μg/mL L-ascorbic acid and 10 mM β-glycerophosphate. Data are expressed as the mean ± SEM. * *p* < 0.05: statistically significant compared to the control. #, *p* < 0.05: statistically significant compared to OS. &, *p* < 0.05: statistically significant compared to the OS + trifol. Data represent the results of three experiments.

The pretreatment of Noggin and PKF118-310 (PKF) significantly abolished Trifol-mediated ALP activity and mineralization during early and late differentiation (Figure 6A,B). On the other hand, pretreatment of SP600125 (SP600) only partially suppressed the Trifol-mediated differentiation (Figure 6A,B). These data suggest that Trifol-stimulated BMP2-mediated Smad1/5/8 and Wnt3-mediated β-catenin signaling pathways promote osteoblast differentiation in osteogenic cells.

## 3. Discussion

In the present study, we investigated the effects of trifol on osteoblast differentiation and maturation. We originally reported novel evidence for bioactive activities related to osteogenic processes via differentiation, migration, and mineralization by a flavonoid *S. flavescens*-derived trifol. The development, formation, remodeling, and repair of bones are mediated by the osteogenic processes involved in osteoblast differentiation [5,30,31,32,33]. Osteoblast dysfunction causes pathological conditions in bone diseases, such as osteoporosis, Paget’s disease, and osteonecrosis [34,35]. Currently, the treatment of bone diseases is limited by side effects and high costs [13,36,37,38]. Several plant-derived compounds have been demonstrated to play a stimulating role in the differentiation and mineralization of osteoblasts, leading to improvement of bone diseases and clinical applications [5,6,37,38,39,40,41,42,43]. Based on the findings of the present study, the flavonoid derived from *S. flavescens*, trifol, promotes osteoblast differentiation and mineralization by increasing osteogenic activity via the stimulation of key proteins (Smad1/5/8 and β-catenin) involved in Wnt3a and BMP2 osteogenic signaling. To date, the intracellular signaling and mechanisms underlying the biological function of trifol in osteogenicity have not been reported. These findings suggest that trifol may be a novel drug source for the treatment of bone diseases such as osteoporosis.

Osteoblast differentiation stimulates the activity and expression of the ALP enzyme, a well-known early osteoblast differentiation marker that induces the hydrolysis of inorganic pyrophosphate and organic phosphomonoesters [5,6,44,45]. With increased ALP activity, collagen and non-collagenous proteins promote calcium deposition and hydroxyapatite crystallization on the extracellular matrix for mineralization and bone formation [46,47]. Our results show that trifol stimulated ALP activity and expression without inducing cytotoxicity. Trifol also upregulated the expression of the osteogenic genes *Alp*, *ColI*, and *Bsp*. RUNX2 is a key regulator of osteogenic processes and increases the expression of various osteogenic genes, including *Alp*, *ColI*, and *Bsp*, leading to the differentiation and maturation of osteogenic cells [48,49,50,51,52,53]. The present study demonstrates that trifol promotes the accumulation of RUNX2 in the nucleus, which aligns with the increased expression of *Alp*, *ColI*, and *Bsp* induced by trifol. These findings suggest that trifol promotes the differentiation of osteogenic cells by increasing ALP activity and mineralized tissue proteins via nuclear RUNX2 accumulation.

Migration phenotypes induce movement into specific niches during osteogenic processes, leading to attachment, osteoid formation, and mineralization [1,54,55,56,57]. The present study demonstrates that trifol accelerates transmigration to the extracellular matrix. The cytoskeletal changes induced by F-actin polymerization promote osteogenic processes [58,59]. In addition, trifol-induced morphological changes are mediated by F-actin polymerization. Osteoblast maturation results in bone matrix mineralization via calcium deposits in the extracellular matrix. Based on our findings, trifol promotes mineralization during osteoblast maturation, which is consistent with the trifol-induced increase in ALP enzyme activity. ALP knockout animals exhibit abnormal mineral apposition and bone fractures [45]. These findings suggest that trifol exerts anabolic activities that accelerate bone formation and mineral apposition.

The differentiation and mineralization of osteoblasts are regulated by RUNX2, which is activated and increased by the osteogenic BMP2, Wnt3, and MAPKs (ERK1/2, JNK, and p38) signaling pathways [60,61]. The binding of BMP2 to BMP2 receptors initiates the canonical Smad1/5/8 and non-canonical MAPKs pathways, and induces RUNX2 activity and expression in the nucleus [62]. The binding of Wnt3a to Frizzled and LRP5/6 receptors induces the degradation of GSK3β and the stabilization of β-catenin, which is translocated into the nucleus and leads to RUNX2 expression in the nucleus [63,64]. Our findings demonstrate that trifol stimulates BMP2 and Wnt3 signaling pathway proteins by promoting Smad1/5/8 phosphorylation, JNK phosphorylation, and β-catenin stabilization. A previous study revealed that trifol induces cytotoxicity and apoptosis of gastric cancer cells by inhibiting the Ras/Raf/MEK/ERK pathway [65]. However, trifol did not affect ERK phosphorylation. The different effects of trifol on ERK could be attributed to the different concentrations and cell types used in the in vitro experimental system. Our data also showed that trifol did not cause cytotoxicity in osteogenic cells, unlike in gastric cancer cells. Therefore, trifol exhibits osteogenic activities by regulating RUNX2 via the stimulation of osteogenic signaling proteins in osteogenic cells. Recently, we reported the osteogenic effects of a secoiridoid glycoside, trifloroside (TriFs) from Gentianae Scabrae Radix roots on osteogenic cells [66]. Compared with trfiol, TriFs has noncytotoxic effects at low concentrations, but increases cell growth at high concen-trations on osteogenic cells. TriFs increased JNK and p38 phosphorylation but not AKT and ERK phosphorylation. TriFs also increased the total expression of RUNX2 in osteo-blast differentiation. Therefore, trifol exhibits osteogenic activity by regulating RUNX2 through stimulation of osteogenic signaling proteins in osteogenic cells in a manner dif-ferent from previously reported trifol.

## 4. Materials and Methods

### 4.1. Plant Material and Isolation

NMR spectroscopy was conducted using a JEOL ECX-500 spectrometer (JEOL Ltd., Tokyo, Japan) operating at ^1^H (500 MHz) and ^13^C (125 MHz). HPLC was performed using an Agilent 1200 Series HPLC system (Agilent Technologies, Santa Clara, CA, USA). Silica gel 60 (70–230 mesh/230–400 mesh ASTM, Merck, Darmstadt, Germany), ODS-A (YMC Co., Ltd., Kyoto, Japan), and Sephadex LH-20 (GE Healthcare, Uppsala, Sweden) were used for column chromatography. *S. flavescens Aiton* was purchased from a commercial herbal medicine company. A voucher specimen was deposited in the Natural Products Bank of the National Institute for Korean Medicine Development (NIKOM, Gyeongsan, Republic of Korea).

To obtain the extract from the roots (1.0 kg), 70% EtOH was used over 3 days (1 × 90 L). The MeOH extract (230.2 g) was evaporated to dryness, suspended in distilled water (7.5 L), and solvent-partitioned using ethyl acetate. The ethyl-acetate-soluble fraction (40.2 g) was subjected to silica gel column chromatography and eluted using a gradient of chloroform, MeOH (40:1 to 0:1, *v*/*v*), to collect 20 fractions (SFCM 1–SFCM 20). Fraction SFCM 7 (2.12 g) was subjected to reverse-phase (ODS-A) column chromatography and eluted with a gradient of MeOH-H_2_O (4:6 to 1:0, *v*/*v*) to collect 10 fractions (SFCM7ME1–SFCM7ME10). Fraction SFCM7ME 3 (820.3 mg) was subjected to Sepadex LH-20 column chromatography and eluted with a gradient of 100% MeOH to obtain trifol (25 mg)

### 4.2. Cell Culture and Trifol Stock Solution

Osteogenic MC3T3E-1 cells (Subclone 4, #CRL-2593) were purchased from American Type Culture Collection (Manassas, VA, USA). The cells were cultured in α-minimum essential medium (α-MEM), without L-ascorbic acid (L-AA), containing 10% FBS and 1X Gibco^®^ Antibiotic-Antimycotic (Thermo Fisher Scientific, Waltham, MA, USA). Differentiation of the osteogenic cells was initiated in an osteogenic medium (OS) containing 10% FBS, 1X Gibco^®^ Antibiotic-Antimycotic (Thermo Fisher Scientific), 50 μg/mL L-AA (Sigma, St. Louis, MO, USA), and 10 mM β-glycerophosphate (Sigma). During the differentiation experiments, the OS was replaced every 2 days. 1000X Trifol stock solution was prepared in 100% dimethyl sulfoxide (DMSO) (Sigma), and 0.1% DMSO was used as the vehicle control.

### 4.3. Cytotoxicity

Cytotoxicity in osteogenic cells was analyzed using an MTT assay. Live cells were treated with MTT solution, and the absorbance of formazan was recorded on a Multiskan GO Microplate Spectrophotometer (Thermo Fisher Scientific) at 540 nm.

### 4.4. ALP Staining and Activity Assays

Osteogenic cells were differentiated for 7 days, and ALP staining and activity were performed as previously described [66].

### 4.5. Total RNA Isolation and RT-PCR Analysis

After osteogenic differentiation for 5 days, the TRIzol reagent (Life Technologies, Gaithersburg, MD, USA) was used to extract the total RNA. Thereafter, reverse transcription of RNA into single-stranded cDNA was performed using AccuPower RT PreMix with oligo (dT)_15_ primers (Bioneer Corporation, Daejeon, Republic of Korea). The band intensities of the PCR products were quantified under ultraviolet exposure (ProteinSimple Inc., Santa Clara, CA, USA) as previously described [67].

### 4.6. Western Blot Analysis

The cells were lysed, and the concentration of the isolated protein was determined using Bradford reagent (Bio-Rad, Hercules, CA, USA). Equal amounts of protein from each sample were separated via SDS-PAGE and transferred onto polyvinylidene fluoride membranes (Millipore, Bedford, MA, USA). After blocking with 5% skim milk in 1×TBS containing 0.05% Tween-20 (TBST), washing with TBST, and incubation with specific primary antibodies overnight at 4 °C, the membranes were washed with TBST and incubated with horseradish-peroxidase-conjugated secondary antibodies (1:10,000; Jackson ImmunoResearch, West Grove, PA, USA). Protein images were monitored using a ProteinSimple Detection System (ProteinSimple Inc., Santa Clara, CA, USA).

### 4.7. Immunofluorescence Assay

Immunofluorescence assays were performed as previously described [68]. The cells were fixed with 4% paraformaldehyde, permeabilized with 0.1% Triton X-100, and blocked with 3% BSA at room temperature. The cells were incubated with anti-RUNX2 antibody (1:200; Cell Signaling Technology, Beverly, MA, USA) overnight at 4 °C, washed, and incubated with Alexa Fluor 488-conjugated secondary antibodies (1:500; Invitrogen, Carlsbad, CA, USA) for 2 h at room temperature. The cells were then co-stained with PI solution (Sigma-Aldrich, St. Louis, MO, USA), and 8-well chamber slides (Thermo Fisher Scientific) were mounted using Fluoromount^TM^ Aqueous Mounting Medium (Sigma-Aldrich). Images were captured using an Olympus IX73 inverted microscope (Olympus Corporation, Tokyo, Japan) and an intravital multi-photon microscope system (IMPM) (Leica Microsystems, Wetzlar, Germany) at the Korea Basic Science Institute (KBSI).

### 4.8. Migration Assay

Cell migration assays were performed using Matrigel-coated membranes in a Boyden chamber as previously described [66]. Matrigel solution (Corning Life Sciences, Tewksbury, MA, USA) and 0.5% crystal violet (Sigma-Aldrich) were used. Images were captured using a light microscope.

### 4.9. Phalloidin and DRAQ5 Staining

F-actin polymerization in osteogenic cells was detected using phalloidin staining (Thermo Fisher Scientific), and the nuclei were detected using DRAQ5 (Thermo Fisher Scientific). The images were captured using an IMPM (Leica Microsystems) at the KBSI.

### 4.10. ARS Staining Assay

Mineralization by calcium deposits in the extracellular matrix was performed using ARS dye, as previously described [66]. After osteogenic cell differentiation was induced, ARS staining was performed, and images of the stained mineralization were observed using a scanner.

### 4.11. Inhibitors

Trifol was treated in the presence or absence of Noggin, PKF118-310 (PKF), and SP600125 (SP600), which are inhibitors against the Smad1/5/8, β-catenin, and JNK pathways, respectively.

### 4.12. Statistical Analysis

The data were analyzed using Prism Version 5 (GraphPad Software Inc., San Diego, CA, USA) and are presented as the mean ± standard error of the mean (SEM). *p* < 0.05 was considered statistically significant, as determined using Student’s unpaired *t*-test.

## 5. Conclusions

In conclusion, *S. flavescens*-derived trifol was found to accelerate osteoblast differentiation and maturation by stimulating osteogenic processes via the osteogenic signaling pathways. Notably, the dysregulation of osteogenic processes leads to abnormal bone formation in bone diseases [34,35]. Although in vivo experiments should be performed to further investigate trifol-induced bone formation, our in vitro data provide new evidence for the use of trifol as a bone-protective compound and bone healing accelerant through its promotion of bone-forming activities.

## Data Availability

The data generated in the current study are available from the corresponding author upon request.

## Abbreviations

ALP	Alkaline phosphatase
ARS	Alizarin Red S.
BMP2	Bone morphogenetic protein 2
BSP	Bone sialoprotein
ColI	Collagen Type I
DMSO	Dimethyl sulfoxide
ECM	Extracellular matrix
HPLC	High performance liquid chromatography
L-AA	L-ascorbic acid
MAPKs	Mitogen-activated protein kinases.
MTT	3-[4,5-dimethylthiazol-2-yl]-2,5-diphenyltetrazolium bromide.
NMR	Nuclear magnetic resonance
OS	Osteogenic supplement medium.
RUNX2	Runt-related transcription factor 2.
TBST	Tris buffered saline, with Tween 20
Trifol TriFs	Trifolirhizin Trifloroside
Wnt3a	Wnt family member 3a

## References

[1] Infante A., Rodriguez C.I. (2018). Osteogenesis and aging: Lessons from mesenchymal stem cells. Stem Cell Res. Ther..

[2] Marie P.J. (2015). Osteoblast dysfunctions in bone diseases: From cellular and molecular mechanisms to therapeutic strategies. Cell Mol. Life Sci..

[3] Fakhry M., Hamade E., Badran B., Buchet R., Magne D. (2013). Molecular mechanisms of mesenchymal stem cell differentiation towards osteoblasts. World J. Stem Cells.

[4] Kobayashi T., Kronenberg H.M. (2021). Overview of Skeletal Development. Methods Mol. Biol..

[5] Yang Y., Zhang T., Jiang M., Yin X., Luo X., Sun H. (2021). Effect of the immune responses induced by implants in a integrated three-dimensional micro-nano topography on osseointegration. J. Biomed. Mater. Res. A.

[6] Park K.R., Kim S., Cho M., Yun H.M. (2021). Limonoid Triterpene, Obacunone Increases Runt-Related Transcription Factor 2 to Promote Osteoblast Differentiation and Function. Int. J. Mol. Sci..

[7] Rosenberg N., Rosenberg O., Soudry M. (2012). Osteoblasts in bone physiology-mini review. Rambam Maimonides Med. J..

[8] Khotib J., Gani M.A., Budiatin A.S., Lestari M., Rahadiansyah E., Ardianto C. (2021). Signaling Pathway and Transcriptional Regulation in Osteoblasts during Bone Healing: Direct Involvement of Hydroxyapatite as a Biomaterial. Pharmaceuticals.

[9] Park K.R., Lee J.Y., Cho M., Hong J.T., Yun H.M. (2021). Biological Mechanisms of Paeonoside in the Differentiation of Pre-Osteoblasts and the Formation of Mineralized Nodules. Int. J. Mol. Sci..

[10] Chen G., Deng C., Li Y.P. (2012). TGF-beta and BMP signaling in osteoblast differentiation and bone formation. Int. J. Biol. Sci..

[11] Martiniakova M., Babikova M., Omelka R. (2020). Pharmacological agents and natural compounds: Available treatments for osteoporosis. J. Physiol. Pharmacol..

[12] Yuan H., Ma Q., Ye L., Piao G. (2016). The Traditional Medicine and Modern Medicine from Natural Products. Molecules.

[13] Gu S.M., Park M.H., Yun H.M., Han S.B., Oh K.W., Son D.J., Yun J.S., Hong J.T. (2016). CCR5 knockout suppresses experimental autoimmune encephalomyelitis in C57BL/6 mice. Oncotarget.

[14] Fabricant D.S., Farnsworth N.R. (2001). The value of plants used in traditional medicine for drug discovery. Environ. Health Perspect..

[15] Sun P., Zhao W., Wang Q., Chen L., Sun K., Zhan Z., Wang J. (2022). Chemical diversity, biological activities and Traditional uses of and important Chinese herb Sophora. Phytomedicine.

[16] Wei G., Chen Y., Guo X., Wei J., Dong L., Chen S. (2021). Biosyntheses characterization of alkaloids and flavonoids in Sophora flavescens by combining metabolome and transcriptome. Sci. Rep..

[17] Zhang J.H., Zhao Y.Y., Liu Q.X., Ye X.J. (2000). Studies on the chemical constituents from Sophora flavescens ait. Zhongguo Zhong Yao Za Zhi.

[18] Zhang L., Xu L., Xiao S.S., Liao Q.F., Li Q., Liang J., Chen X.H., Bi K.S. (2007). Characterization of flavonoids in the extract of Sophora flavescens Ait. by high-performance liquid chromatography coupled with diode-array detector and electrospray ionization mass spectrometry. J. Pharm. Biomed. Anal..

[19] Huang R., Liu Y., Zhao L.L., Chen X.X., Wang F., Cai W., Chen L. (2017). A new flavonoid from Sophora flavescens Ait. Nat. Prod. Res..

[20] Huang X.B., Yuan L.W., Shao J., Yang Y., Liu Y., Lu J.J., Chen L. (2021). Cytotoxic effects of flavonoids from root of Sophora flavescens in cancer cells. Nat. Prod. Res..

[21] Jin J.H., Kim J.S., Kang S.S., Son K.H., Chang H.W., Kim H.P. (2010). Anti-inflammatory and anti-arthritic activity of total flavonoids of the roots of Sophora flavescens. J. Ethnopharmacol..

[22] Yang X., Yang J., Xu C., Huang M., Zhou Q., Lv J., Ma X., Ke C., Ye Y., Shu G. (2015). Antidiabetic effects of flavonoids from Sophora flavescens EtOAc extract in type 2 diabetic KK-ay mice. J. Ethnopharmacol..

[23] Liu Y., Zeng W., Ma C., Wang Z., Wang C., Li S., He W., Zhang Q., Xu J., Zhou C. (2020). Maackiain dampens osteoclastogenesis via attenuating RANKL-stimulated NF-kappaB signalling pathway and NFATc1 activity. J. Cell Mol. Med..

[24] Chiou W.F., Lee C.H., Liao J.F., Chen C.C. (2011). 8-Prenylkaempferol accelerates osteoblast maturation through bone morphogenetic protein-2/p38 pathway to activate Runx2 transcription. Life Sci..

[25] Sun D., Tao W., Zhang F., Shen W., Tan J., Li L., Meng Q., Chen Y., Yang Y., Cheng H. (2020). Trifolirhizin induces autophagy-dependent apoptosis in colon cancer via AMPK/mTOR signaling. Signal Transduct. Target. Ther..

[26] Zhang Q., Wang S., Ji S. (2022). Trifolirhizin regulates the balance of Th17/Treg cells and inflammation in the ulcerative colitis mice through inhibiting the TXNIP-mediated activation of NLRP3 inflammasome. Clin. Exp. Pharmacol. Physiol..

[27] Zhou H., Lutterodt H., Cheng Z., Yu L.L. (2009). Anti-Inflammatory and antiproliferative activities of trifolirhizin, a flavonoid from Sophora flavescens roots. J. Agric. Food Chem..

[28] Aratanechemuge Y., Hibasami H., Katsuzaki H., Imai K., Komiya T. (2004). Induction of apoptosis by maackiain and trifolirhizin (maackiain glycoside) isolated from sanzukon (Sophora Subprostrate Chen et T. Chen) in human promyelotic leukemia HL-60 cells. Oncol. Rep..

[29] Hyun S.K., Lee W.H., Jeong D.M., Kim Y., Choi J.S. (2008). Inhibitory effects of kurarinol, kuraridinol, and trifolirhizin from Sophora flavescens on tyrosinase and melanin synthesis. Biol. Pharm. Bull..

[30] Histing T., Stenger D., Kuntz S., Scheuer C., Tami A., Garcia P., Holstein J.H., Klein M., Pohlemann T., Menger M.D. (2012). Increased osteoblast and osteoclast activity in female senescence-accelerated, osteoporotic SAMP6 mice during fracture healing. J. Surg. Res..

[31] Zayzafoon M. (2006). Calcium/calmodulin signaling controls osteoblast growth and differentiation. J. Cell Biochem..

[32] Broz A., Ukraintsev E., Kromka A., Rezek B., Hubalek Kalbacova M. (2017). Osteoblast adhesion, migration, and proliferation variations on chemically patterned nanocrystalline diamond films evaluated by live-cell imaging. J. Biomed. Mater. Res. A.

[33] Tome M., Lopez-Romero P., Albo C., Sepulveda J.C., Fernandez-Gutierrez B., Dopazo A., Bernad A., Gonzalez M.A. (2011). miR-335 orchestrates cell proliferation, migration and differentiation in human mesenchymal stem cells. Cell Death Differ..

[34] Deng T., Zhang W., Zhang Y., Zhang M., Huan Z., Yu C., Zhang X., Wang Y., Xu J. (2021). Thyroid-stimulating hormone decreases the risk of osteoporosis by regulating osteoblast proliferation and differentiation. BMC Endocr. Disord..

[35] Shalehin N., Hosoya A., Takebe H., Hasan M.R., Irie K. (2020). Boric acid inhibits alveolar bone loss in rat experimental periodontitis through diminished bone resorption and enhanced osteoblast formation. J. Dent. Sci..

[36] Mishra B.B., Tiwari V.K. (2011). Natural products: An evolving role in future drug discovery. Eur. J. Med. Chem..

[37] An J., Yang H., Zhang Q., Liu C., Zhao J., Zhang L., Chen B. (2016). Natural products for treatment of osteoporosis: The effects and mechanisms on promoting osteoblast-mediated bone formation. Life Sci..

[38] Soelaiman I.N., Das S., Shuid A.N., Mo H., Mohamed N. (2013). Use of medicinal plants and natural products for treatment of osteoporosis and its complications. Evid. Based Complement. Alternat Med..

[39] Liang J., Bao A.L., Ma H.Y., Dong W., Li W.H., Wu X., Li H.Y., Hou H.Y., Chen Y.Q., Fu J.L. (2022). Prevention of polycystic ovary syndrome and postmenopausal osteoporosis by inhibiting apoptosis with Shenling Baizhu powder compound. PeerJ.

[40] Xue C., Pan W., Lu X., Guo J., Xu G., Sheng Y., Yuan G., Zhao N., Sun J., Guo X. (2021). Effects of compound deer bone extract on osteoporosis model mice and intestinal microflora. J. Food Biochem..

[41] Di Y., Wasan E.K., Cawthray J., Syeda J., Ali M., Cooper D.M.L., Al-Dissi A., Ashjaee N., Cheng W., Johnston J. (2021). Evaluation of La(XT), a novel lanthanide compound, in an OVX rat model of osteoporosis. Bone Rep..

[42] Li M., Shi X.L., Xu C., Wu L.G., He B., Li Y.H., Liang B.C. (2020). Mechanism action of Chinese herbal compound and target network pharmacology of Yougui (YG) pill for the treatment of osteoporosis. Zhongguo Gu Shang.

[43] Bernardini S., Tiezzi A., Laghezza Masci V., Ovidi E. (2018). Natural products for human health: An historical overview of the drug discovery approaches. Nat. Prod. Res..

[44] Orimo H. (2010). The mechanism of mineralization and the role of alkaline phosphatase in health and disease. J. Nippon. Med. Sch..

[45] Wennberg C., Hessle L., Lundberg P., Mauro S., Narisawa S., Lerner U.H., Millan J.L. (2000). Functional characterization of osteoblasts and osteoclasts from alkaline phosphatase knockout mice. J. Bone Miner. Res..

[46] Lin X., Patil S., Gao Y.G., Qian A. (2020). The Bone Extracellular Matrix in Bone Formation and Regeneration. Front. Pharmacol..

[47] Ogata Y. (2008). Bone sialoprotein and its transcriptional regulatory mechanism. J. Periodontal Res..

[48] Amarasekara D.S., Kim S., Rho J. (2021). Regulation of Osteoblast Differentiation by Cytokine Networks. Int. J. Mol. Sci..

[49] Schroeder T.M., Jensen E.D., Westendorf J.J. (2005). Runx2: A master organizer of gene transcription in developing and maturing osteoblasts. Birth Defects Res. C Embryo Today.

[50] Komori T. (2010). Regulation of bone development and extracellular matrix protein genes by RUNX2. Cell Tissue Res..

[51] Huang R.L., Yuan Y., Tu J., Zou G.M., Li Q. (2014). Opposing TNF-alpha/IL-1beta- and BMP-2-activated MAPK signaling pathways converge on Runx2 to regulate BMP-2-induced osteoblastic differentiation. Cell Death Dis..

[52] Yun H.M., Park K.R., Quang T.H., Oh H., Hong J.T., Kim Y.C., Kim E.C. (2015). 2,4,5-Trimethoxyldalbergiquinol promotes osteoblastic differentiation and mineralization via the BMP and Wnt/beta-catenin pathway. Cell Death Dis..

[53] Phimphilai M., Zhoa Z.R., Boules H., Roca H., Franceschi R.T. (2006). BMP signaling is required for RUNX2-dependent induction of the osteoblast phenotype. J. Bone Miner. Res..

[54] Granero-Molto F., Weis J.A., Miga M.I., Landis B., Myers T.J., O’Rear L., Longobardi L., Jansen E.D., Mortlock D.P., Spagnoli A. (2009). Regenerative effects of transplanted mesenchymal stem cells in fracture healing. Stem Cells.

[55] Delaisse J.M. (2014). The reversal phase of the bone-remodeling cycle: Cellular prerequisites for coupling resorption and formation. Bonekey Rep..

[56] Kalbacova M., Broz A., Kong J., Kalbac M. (2010). Graphene substrates promote adherence of human osteoblasts and mesenchymal stromal cells. Carbon.

[57] Aryaei A., Jayatissa A.H., Jayasuriya A.C. (2014). The effect of graphene substrate on osteoblast cell adhesion and proliferation. J. Biomed. Mater. Res. Part A.

[58] Tong Z., Liu Y., Xia R., Chang Y., Hu Y., Liu P., Zhai Z., Zhang J., Li H. (2020). F-actin Regulates Osteoblastic Differentiation of Mesenchymal Stem Cells on TiO2 Nanotubes Through MKL1 and YAP/TAZ. Nanoscale Res. Lett..

[59] Xue X., Hong X., Li Z., Deng C.X., Fu J. (2017). Acoustic tweezing cytometry enhances osteogenesis of human mesenchymal stem cells through cytoskeletal contractility and YAP activation. Biomaterials.

[60] Wozney J.M., Rosen V., Celeste A.J., Mitsock L.M., Whitters M.J., Kriz R.W., Hewick R.M., Wang E.A. (1988). Novel regulators of bone formation: Molecular clones and activities. Science.

[61] Krishnan V., Bryant H.U., MacDougald O.A. (2006). Regulation of bone mass by Wnt signaling. J. Clin. Investig..

[62] Canalis E., Economides A.N., Gazzerro E. (2003). Bone morphogenetic proteins, their antagonists, and the skeleton. Endocr. Rev..

[63] Reya T., Clevers H. (2005). Wnt signalling in stem cells and cancer. Nature.

[64] Gaur T., Lengner C.J., Hovhannisyan H., Bhat R.A., Bodine P.V., Komm B.S., Javed A., van Wijnen A.J., Stein J.L., Stein G.S. (2005). Canonical WNT signaling promotes osteogenesis by directly stimulating Runx2 gene expression. J. Biol. Chem..

[65] Lu X., Ma J., Qiu H., Yang L., Cao L., Shen J. (2016). Anti-proliferation effects of trifolirhizin on MKN45 cells and possible mechanism. Oncol. Rep..

[66] Yun H.M., Kim B., Park J.E., Park K.R. (2022). Trifloroside Induces Bioactive Effects on Differentiation, Adhesion, Migration, and Mineralization in Pre-Osteoblast MC3T3E-1 Cells. Cells.

[67] Yun H.M., Kim B., Jeong Y.H., Hong J.T., Park K.R. (2023). Suffruticosol A elevates osteoblast differentiation targeting BMP2-Smad/1/5/8-RUNX2 in pre-osteoblasts. Biofactors.

[68] Park K.R., Park J.E., Kim B., Kwon I.K., Hong J.T., Yun H.M. (2021). Calycosin-7-O-beta-Glucoside Isolated from Astragalus membranaceus Promotes Osteogenesis and Mineralization in Human Mesenchymal Stem Cells. Int. J. Mol. Sci..

